# Local and Regional Determinants of Colonisation-Extinction Dynamics of a Riparian Mainland-Island Root Vole Metapopulation

**DOI:** 10.1371/journal.pone.0056462

**Published:** 2013-02-20

**Authors:** Petter Glorvigen, Harry P. Andreassen, Rolf A. Ims

**Affiliations:** 1 Faculty of Applied Ecology and Agricultural Sciences, Hedmark University College, Koppang, Norway; 2 Department of Arctic and Marine Biology, University of Tromsø, Tromsø, Norway; University of Western Ontario, Canada

## Abstract

The role of local habitat geometry (habitat area and isolation) in predicting species distribution has become an increasingly more important issue, because habitat loss and fragmentation cause species range contraction and extinction. However, it has also become clear that other factors, in particular regional factors (environmental stochasticity and regional population dynamics), should be taken into account when predicting colonisation and extinction. In a live trapping study of a mainland-island metapopulation of the root vole (*Microtus oeconomus*) we found extensive occupancy dynamics across 15 riparian islands, but yet an overall balance between colonisation and extinction over 4 years. The 54 live trapping surveys conducted over 13 seasons revealed imperfect detection and proxies of population density had to be included in robust design, multi-season occupancy models to achieve unbiased rate estimates. Island colonisation probability was parsimoniously predicted by the multi-annual density fluctuations of the regional mainland population and local island habitat quality, while extinction probability was predicted by island population density and the level of the recent flooding events (the latter being the main regionalized disturbance regime in the study system). Island size and isolation had no additional predictive power and thus such local geometric habitat characteristics may be overrated as predictors of vole habitat occupancy relative to measures of local habitat quality. Our results suggest also that dynamic features of the larger region and/or the metapopulation as a whole, owing to spatially correlated environmental stochasticity and/or biotic interactions, may rule the colonisation – extinction dynamics of boreal vole metapopulations. Due to high capacities for dispersal and habitat tracking voles originating from large source populations can rapidly colonise remote and small high quality habitat patches and re-establish populations that have gone extinct due to demographic (small population size) and environmental stochasticity (e.g. extreme climate events).

## Introduction

The dynamics of metapopulations are driven by the relative probabilities for colonisation and extinction among local habitat patches and populations. These probabilities depend on (1) characteristics innate to the species, such as dispersal ability and local demographic processes, (2) to characteristics of the local habitat patches such as their geometry (size and isolation) and quality (carrying capacity), and (3) to regional factors due to large-scale stochastic events and ecological dynamics. MacArthur and Wilson [1] pioneered the study of extinction-colonisation dynamics by their theory of island biography which derived how species occupancy through rates of colonisation and extinction were functions of habitat geometric parameters such as island size and island isolation (from other islands and the mainland). Similarly, in metapopulation theory, that largely has replaced the island biography theory as the main framework for predicting species habitat occupancy, habitat area and isolation are the single two factors influencing the probabilities of local re-colonisation and extinction and likelihood of persistence of the metapopulation as a whole [2]. Over the years it has been clear that local population size and habitat quality may have strong independent effects that may be larger than habitat area in predicting local population extinction, and habitat quality may be better than habitat area in predicting colonisation [3], [4], [5], [6], [7], [8], [9], [10], [11]. Moreover, habitat isolation may have little predictive power regarding colonisation compared to habitat quality [10]. This occurs even when isolation is carefully calculated as a patch-specific connectivity metric in terrestrial landscapes, combining attributes of the landscape matrix and specific traits of the focal species [9], [12]. One may argue that if the study scale was small or the matrix was easily traversed relative to the dispersal capacity of the focal species the effect of isolation may be negligible (sensu effective isolation in Ricketts [13] and the concept of patchy populations in Harrison [14]). In particular, species with high dispersal capacity may prospect most patches available within the metapopulation and select those patches that best suit their specific habitat requirements for settlement [11].

The importance of patch area and isolation may also diminish in the presence of disproportionally large high quality patches (or a mainland) that can harbour source populations that either constantly or periodically provide a flow of migrants that cause colonisation of all high quality habitats irrespectively of their isolation [15], [16]. Such a situation has in the metapopulation literature been described as mainland-island and/or source-sink metapopulations [2]. Another important circumstance is spatially correlated fluctuations in the system, for instance, caused by environmental stochasticity or large-scale biotic dynamics that may both impinge on the quality of local patches (e.g. affecting local extinction probabilities [17]) or the quality of the matrix (e.g. affecting the pool of migrants and their success [18]).

Small mammals, and in particular arvicoline rodents such as voles, are well known for their often violent spatio-temporal population dynamics, with frequent local colonisation and extinction events, but also with a profound large-scale component in terms of regionalized multi-annual population fluctuations. Their spatial distribution is commonly described as patchy [19]. It is assumed that local populations persist only in high quality patches during regional population lows, while re-colonisation of patches of lower quality explains their wider distribution during regional population peak [20].

Vole species have been advocated to be among the best suited models for investigating the relative importance of local habitat heterogeneity and the impact of more large-scale drivers on spatio-temporal dynamics [19], [21], [22], [23], [24]. Still the key parameters of metapopulations (i.e. extinction and colonisation) as functions of ecologically relevant factors have been rarely estimated for voles (for an exception see [25] that however did not correct for detection probability). In this paper we report from a 4-year study of metapopulation dynamics of the root vole *Microtus oeconomus* in a riparian island system. Our main aim is to explore the predictive powers of local (island-specific) factors relative to regional predictors of colonisation and extinction probabilities. Among local (island-specific) predictors we considered habitat size and isolation, habitat quality and habitat patch-specific population density, whereas as regional predictors we considered stochastic flooding events as well as multi-annual population fluctuations. The root vole is a species suitable for this purpose as it can be considered to be a habitat specialist with fairly well-known habitat preferences [26] so that potential habitats within the metapopulation could be defined and habitat quality be assessed. It is also known to show strong temporal population fluctuations due to both regional biotic and abiotic factors [27], [28], [29], [30], [31]. As the root vole is a secretive organism [32] we have taken into account the probability of detection (i.e. causing false non-occupancy and pseudo-extinctions) in our estimates of colonisation and extinction.

## Materials and Methods

### Ethics Statement

The study was approved by The Norwegian Directorate for Nature Management (2007/11612 ART-VI-ID and 2009/3107 ART-VI-ID) and followed the current laws in Norway (animal welfare; LOV-2009-06-19-97, capture of wild animals for scientific purposes; FOR 2003-03-14 nr 349). Access to the study area was approved by the private landowners. The way of trapping voles employed (i.e. regular trap checks and releasing individuals on spot) is not considered as an animal experiment and therefore requires no license from the Norwegian Animal Experiment Board. In order to secure fast and correct handling of voles experienced field personnel conducted the trap checks. All captured individuals were released after a few minutes of handling. Other species captured in this study were released on spot and none of these species are protected in Norway or included on the Norwegian Red list.

### Study Area and Field Methods

**Figure 1:**
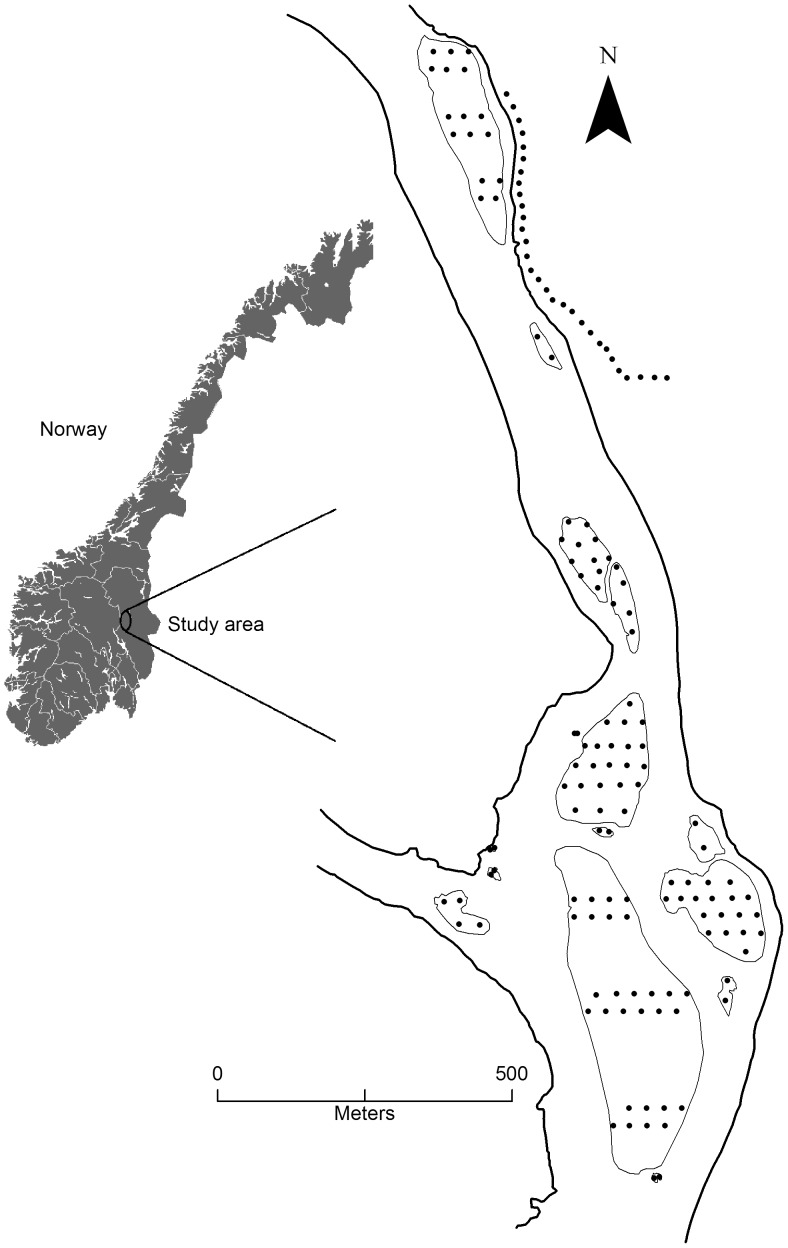
Map of study location in Norway and the 15 islands in the river Glomma. Black dots represent trap locations on the islands and on the mainland.

The 5 km^2^ study area in Østerdalen, Hedmark county, Norway (61°54′ N, 11°04′ E) consisted of 15 islands in Glomma which is the largest river in Norway (Figure 1). The surrounding landscape consists of a mosaic of agricultural areas, commercially managed forest stands and scattered human settlements. In the study area the water level of the river peaks during snow melt in the northern mountainous catchment area and during occasional heavy rainfalls in the snow free season. During the study (2008–2011) the mean summer water flow was approximately 1400 cubic meter per second and the water level varied between 253.8 meters and 257.7 meters above sea level (asl), thus representing a dynamic component of assumed ecological importance, especially as the islands are flat and thus readily flooded. The 15 islands represent all islands in a cluster with relatively short inter-patch distance compared to up- and downstream islands outside the study area. The nearest island outside the cluster is approximately 0.5 km upstream and was considered to be too far away to influence on the rodent dynamics within our selected cluster of islands. All 15 islands were permanently isolated by water during the study. Because the available maps were inaccurate and the varying water level influenced on island size and distance measurements, all island related measurements (e.g. perimeter and distance between islands) were gathered by hand-held GPS units at low water level (254.8 m asl, 2^nd^ of July 2008). Water level measurements were provided by the Norwegian Water Resources and Energy Directorate (NVE), collected every second hour all year round at Stai gauging station ∼ 4 kilometres downstream. Map construction and measurement calculations were done in ArcMap 10 (Environmental Systems Research Institute, Inc. 1999).

We live trapped voles on all islands by regularly spaced Ugglan traps (Grahnab, Marieholm, Sweden) baited with carrot, oat and sun flower seeds. We aimed at a 30 m×30 m spacing between all traps, except on the two largest islands which had less traps (Figure 1). On the smaller island a minimum of two traps were used. To provide data on the influence of mainland populations (i.e. as a potentially regional source population with multi-annual fluctuations) we also monitored voles along the eastern mainland shore by means of trap-line consisting of 30 traps with 30 m spacing (Figure 1). Each year we conducted 2–5 trapping sessions (hereafter termed seasons), each consisting of 3–5 trap checks (hereafter termed surveys). The time and duration of a trapping session could not be exactly planned as strong currents at high water levels prevented safe boat trips. The status of root voles on each island was classified as either not detected (0) or detected (1), adding up to an detection history consisting of 54 surveys distributed on 13 seasons (Table 1). The islands were considered as closed regarding colonisation (immigration) and extinction (emigration) during a survey (2–3 days of trapping) and open to colonisation (immigration) and extinction (emigration) between seasons (minimum 25 days), similar to a robust design [33]. Our study design with the use of traps rules out the possibility of false detection, and detection of voles on one island is independent of detection on all other islands [34], [35]. All captured animals were individually marked (toe clipping) and accounted for only once during a season (i.e. when captured in more than one survey within season). All traps and remaining bait were removed from the islands and the mainland between seasons.

**Table 1:** Time schedule of the study and trapping results.

Year	Week	Number of surveys	Islands occupied	Islands colonized	Islands extinct	Individuals mainland	Individuals islands
2008	27	5	6			9	20
2008	31	5	10	4	0	8	15
2008	35	5	11	1	0	0	6
2008	40	4	8	1	4	1	2
2009	23	4	2	0	6	4	1
2009	27	5	5	3	0	0	20
2009	31	5	3	0	2	1	20
2009	35	5	4	1	0	2	19
2009	39	4	6	2	0	10	20
2010	24	4	6	3	3	22	95
2010	37	3	13	7	0	6	179
2011	23	3	9	0	4	8	69
2011	36	3	6	3	6	0	46

### Measuring Covariates of Island Occupancy

We considered five categories of predictors with *a priori* expected influence on island occupancy in root voles (see table 2 for predictions for the different covariates). 1) *Island geometry* predictors were island perimeter as indices of island size (PERIM; range: 27–1315 meters), and as two indices of island isolation we measured distance from an island to the nearest mainland shore (DISTML; range: 5–153 meters) and the distance from an island to the nearest upstream island or (if nearer) mainland source population the previous season (DISTS; range: 5–153 meters). Only upstream sources were considered because we deemed it impossible for voles to swim against the currents. Similarly unpredictable “swimming” range estimates in the occurrence of strong currents hampered calculations of a connectivity measure [12]. Population size of neighbours and the average population size on all islands were also tried as connectivity predictors but did not improve the models. 2) *Habitat quality* was indexed at every trap location but aggregated to the island level for the analyses. The proportion of different field layer vegetation species expected to provide cover or food resources for root voles were estimated in a 1 m^2^ sampling plot encompassing the trap. The summed proportion of grass, forb and berry producing shrubs over all plots divided by the number of plots/traps on the island was used as index for the island specific quantity of food and cover (PFOOD). PFOOD was strongly positively correlated with tree-layer measurements (number of trees, tree species and tree height were registered within a 50 m^2^ circle of the trap) so PFOOD was used as the only predictor of habitat quality. 3) *River dynamics* (and thus the extent of flooding) were described with two predictors: minimum and maximum water level (WMIN and WMAX) between two seasons (i.e. the period open to colonisation and extinction). 4) *Population density* was indexed seasonally both for the mainland (POPML) and for each island (POPISL). The population density index was calculated as the number of unique individuals captured in a given season, divided by the number of traps and trap checks on the island (the latter to account for the fact that the number of trap checks varied between seasons). In the models of occupancy dynamics (see below) the previous season’s population density indices were entered the models. 5) *Season* was taken as the week number of the year and was considered both in a linear (WEEK) and a quadratic form (WEEK∧2) because the occupancy dynamics could depend on the overall abundance of voles which either was expected to show a seasonal increase towards autumn (week) or a summer peak (week∧2).

**Table 2:** Estimates ± SE on the logit scale for the covariates used to model the occupancy dynamics (ψ_0_, γ, ε, *p*) of the root vole metapopulation.

			Island geometry			Habitat quality	River dynamics		Population density		Season	
Parameter	Model	Intercept	PERIM	DISTML	DISTS	PFOOD	WMAX	WMIN	POPML	POPISL	WEEK	WEEK∧2
	Predicted		+	–		+						
**Occupancy (ψ_0_)**	Best (SE)	–0.96(0.90)				2.46(1.16)						
	Univariate (SE)	NA	1.27(0.79)	–0.47(0.66)		2.48(1.17)*						
	Predicted		+	–	–	+	–	–	+		+	Summer peak
**Colonisation (γ)**	Best (SE)	–0.64(0.32)				1.11(0.39)			1.49(0.46)			
	Univariate (SE)	NA	0.30(0.47)	–0.05(0.24)	–0.48(0.36)	0.67(0.29)*	–0.18(0.25)	–0.14(0.24)	1.10(0.32)*		0.37(0.27)	–0.98(3.58)
	Predicted		–	+	+	–	+	+	–	–	–	Summer low
**Extinction (ε)**	Best (SE)	–0.69 (0.63)	NA			NA	1.80(0.59)			–0.71(0.30)		NA
	Univariate (SE)	NA	–0.79(0.29)*	–0.13(0.25)	–0.08(0.21)	–0.65(0.27)*	0.91(0.29)*	–0.41(0.29)	–0.33(0.28)	–0.65(0.26)*	–0.41(0.23)	6.29(2.72)*
	Predicted		+			+					+	Summer peak
**Detectection (** ***p*** **)**	Best (SE)	0.60(0.14)	0.63(0.12)			NA					NA	–6.30(1.47)
	Univariate (SE)	NA	0.69(0.13)*			0.77(0.15)*					–0.45(0.14)*	–3.83(1.66)*

NOTE: Estimates from univariate models used to identify covariates for the multivariate analyses and from the best multivariate model are shown. Provided is also the sign of the a priori predicted effects. All covariates in the best model had at strong impact, i.e. 95% CI (*β* ±1.96xSE) do not overlap 0; * denotes covariates with strong univariate impact and thus included in the multivariate models; PERIM = perimeter; DISTML = distance to mainland; DISTS = distance to nearest source population; PFOOD = proportion of food and cover; WMAX = maximum water level; WMIN = minimum water level; POPML = population density index for the mainland the previous season; POPISL = population density index for the island the previous season; WEEK = week of the year; WEEK∧2 = week of the year on a quadratic form.

Time intervals between seasons and whether the season included ice on the river (i.e. in winter) were also considered as covariates. Both of these variables were however strongly correlated with water level (WMAX) and could not be included together with this variable in multivariate models (see below).

### Analysing Occupancy Dynamics

We used the program PRESENCE 4.3 to derive the multi-seasonal occupancy dynamics and the associated covariate impact from the sampled detection histories [36]. This modelling approach provides unbiased estimates of the probability of occupancy (ψ), colonisation (γ) and extinction (ε) when the probability of detecting present individuals in a population (*p*) is less than one [34], [35]. PRESENCE uses the observed detection history for a site over a series of surveys (1 = presence, 0 = not detected). The probability of not detecting voles arises from two possible events: either voles were there, but were never detected, or voles were genuinely absent from the island. By combining probabilistic statements for all islands, maximum likelihood estimates of the model parameters can be obtained. The model also enables parameters to be function of covariates. Predictors are entered into the model by way of the logistic model (logit link) [36]. Our aim was to evaluate the explanatory power of the different predictors on colonisation and extinction probabilities, while at the same time accounting for covariates related to initial island occupancy (ψ_0_ = island occupancy in year 2008) and heterogeneity in detection probabilities (*p*) between islands and seasons (the multi-season parameterization in PRESENCE, *sensu* MacKenzie [34]). Colonisation probability (γ) is the probability that an unoccupied island at season *t* is occupied at season *t* +1. Extinction probability (ε) is the probability that an occupied island at season *t* is extirpated at season *t* +1. To avoid a large set of candidate models with covariates of little (or none) explanatory power, we applied a two-stage approach to identify covariates with estimates indicating strong and robust impact, i.e. estimates with 95% CI (*β* ±1.96 * SE) not overlapping 0 [37], [38]. In the first stage, we used univariate models for initial evaluation of the relationship between covariates and the rate parameters (ψ_0_, γ, ε, and *p*). For each evaluation of covariate impact on one parameter, all other parameters were held constant. The univariate models of initial occupancy (ψ_0_) consisted of PFOOD and the island geometrical covariates, except from DISTS which was obviously not known in advance of the study. For the same reason population size and river dynamics could not be evaluated as covariates of initial occupancy. The detection probability (*p*) may be closely related to population size [39]. However, estimates of population size depend on detection and cannot be used as covariates modelling detection, i.e. they are circular [35]. Nevertheless, the covariates PERIM, PFOOD and the seasonal are likely to function as proxies for population size in the univariate models of detection probability. The covariates island geometry, habitat quality, river dynamics and season were all evaluated in univariate models of colonisation probability (γ) and extinction probability (ε). The impact of POPML was evaluated for colonisation probability, and both indices of population density were evaluated for extinction probability. The *a priori* predicted sign of each covariate impact is specified in Table 2. Covariates were standardized (Z scores) before inclusion in the models when necessary.

In the second stage, covariates with a strong indication of impact in univariate models on the rate parameters (ψ_0_, γ, ε, and *p*) were included in the construction of additive multivariate models. All combinations containing at least one of the covariates were evaluated. Covariates with a Pearson correlation coefficient >0.60 were not added simultaneously as covariates to the same rate parameter. We used AIC, ΔAIC, and AIC weights when selecting the best model for inference of covariate impact [40].

## Results

### Raw Occupancy History and Univariate Modelling

Fourteen out of the 15 islands were occupied at least in one season by root voles. One island was occupied in all seasons. Modelling all the parameters as constants, i.e. not restricting the detection history to be a function of a covariate, showed that the estimated average detection probability was 0.70±0.03 (estimate ± SE), demonstrating the necessity to account for imperfect detection. Initial occupancy was estimated to 0.40 (±0.13 SE). The number of islands occupied ranged from 2 to 13, and we registered 25 colonisation events and 25 extinction events (Table 1). Colonisation balanced extinctions, thus the metapopulation could be considered to be stationary, and the estimated equilibrium occupancy from the raw occupancy history was 0.53 (±0.07 SE). Mainland population size varied extensively over the 13 seasons (see Table 1) and was correlated with the total population size for all islands the current season (Pearson correlation coefficient = 0.40) and even more so the following season (Pearson correlation coefficient = 0.86).

The univariate modelling approach indicated that all parameters (ψ_0_, γ, ε, p) were better modelled as a function of at least one predictor (Table 2). Univariate modelling of initial occupancy gave no evidence for effects of any island geometry predictors (±1.96*SE>*β*). Colonisation probability was neither significantly affected by island geometry, river dynamics or season. DISTML, DISTS, WMIN, POPML and WEEK had little impact on extinction probability. All covariates with strong impact on rate parameters are shown in Table 2 and the sign of their effects were all as *a priori* expected.

### Inferences from Multivariate Modelling of Island Occupancy Dynamics

The multivariate occupancy analyses showed that the top ranking model had strong support compared to the second best model (ΔAIC = 4.05; Table 3). The most parsimonious model accounted for 82% of the AIC model weights. The difference in included covariates between the top ranking model and the second best model consisted of a replacement of POPISL with PERIM as covariate of the extinction probability. Because larger islands tended to have higher values of the population density index in most seasons (Pearson correlation >0.60 in 8 out of the 13 seasons) POPISL and PERIM was not included simultaneously as covariates for the same rate parameter. Based on ΔAIC, population density (POPISL) was better than island perimeter (PERIM) in terms of predicting extinction.

**Table 3:** The 10 top ranking models depicting initial island occupancy (ψ_0_), colonisation (γ), extinction (ε) and detection (*p*).

Model	AIC	ΔAIC	ω*_i_*	K
ψ_0_(PFOOD), γ(POPML+PFOOD), ε(POPISL+WMAX), p(PERIM+TIME∧2)	561.45	0.00	0.82	12
ψ_0_(PFOOD), γ(POPML+PFOOD), ε(PERIM+WMAX), p(PERIM+TIME∧2)	565.50	4.05	0.11	12
ψ_0_(PFOOD), γ(POPML), ε(POPISL+WMAX), p(PERIM+TIME∧2)	568.51	7.06	0.02	11
ψ_0_(.), γ(POPML+PFOOD), ε(POPISL+WMAX), p(PERIM+TIME∧2)	568.97	7.52	0.02	11
ψ_0_(PFOOD), γ(POPML+PFOOD), ε(PFOOD+WMAX), p(PERIM+TIME∧2)	570.31	8.86	0.01	12
ψ0(PFOOD), γ(POPML+PFOOD), ε(POPISL+WMAX), p(PFOOD+TIME∧2)	571.00	9.55	0.01	12
ψ_0_(PFOOD), γ(POPML+PFOOD), ε(POPISL+WMAX), p(PERIM+TIME)	571.54	10.09	0.01	11
ψ_0_(PFOOD), γ(POPML), ε(PERIM+WMAX), p(PERIM+TIME∧2)	572.64	11.19	0.00	11
ψ_0_(.), γ(POPML+PFOOD), ε(PERIM+WMAX), p(PERIM+TIME∧2)	573.03	11.58	0.00	11
ψ_0_(PFOOD), γ(POPML+PFOOD), ε(PERIM+WMAX), p(PERIM+TIME)	573.75	12.30	0.00	11

NOTE: models are ranked according to Akaike’s Information Criterion (AIC) and differences in AIC (ΔAIC). ωi = AIC weights; K = number of parameters; for definition of covariates see table 2 and Methods.

All covariate estimates of the best model showed a strong impact on the associated rate parameter (Table 2). Islands with higher proportions of the food and cover vegetation preferred by root voles (PFOOD) were more likely to be occupied the first season of the study (Table 2). Occupied islands had on average 50.60% (±4.62 SE) coverage of food and cover species, while the unoccupied islands had only 19.40% (±5.38 SE).

#### Probability of detection

**Figure 2:**
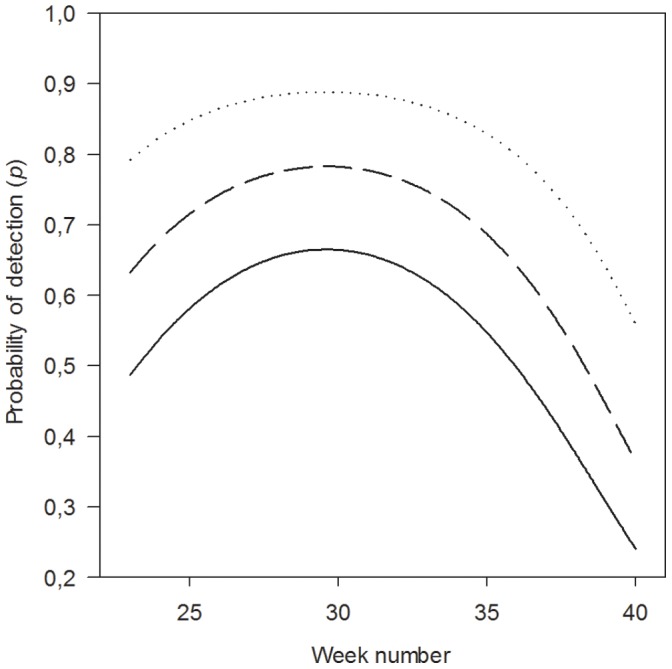
Probability of detection. The relationship between the probability of detection (*p*), week of the year (WEEK∧2) and island perimeter (PERIM; 40 m = solid line, 358 m = broken line, 800 m = dotted line).

The probability of detection was positively related to the perimeter of islands and varied with season (i.e. PERIM and WEEK∧2; Table 2). Detection probability peaked in the middle of summer (∼ week 30). In week 30 the estimated detection probability was <0.67 on the two smallest islands (perimeter <40 m), 0.78 on medium sized islands (perimeter = 358 m), and >0.88 on the two largest islands (perimeter >800 m; Figure 2).

#### Probability of colonisation

**Figure 3:**
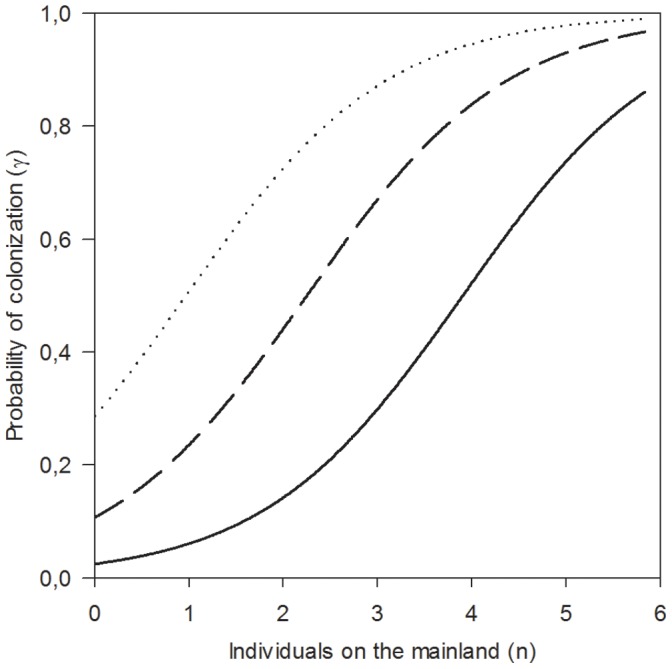
Probability of colonisation. The relationship between the probability of colonisation (γ), population density index on the mainland the previous season (POPML) and proportion of food and cover on the ground (PFOOD; 5% = solid line, 33% = broken line and 55% = dotted line).

The probability of colonisation was positively related to the proportion of the preferred food and cover vegetation (PFOOD) and to mainland population density the previous season (POPML; Table 2). Following a season with a medium density of voles on the mainland (INDVML = 2.5) the probability of colonisation was <0.21 on the two islands with the lowest proportions of the preferred vegetation (PFOOD <5%). In comparison the probability of colonisation was 0.56 on islands with an average proportion of food and cover vegetation (PFOOD = 33%), while on the two islands with the highest proportions of food and cover (PFOOD >55%) the probability of colonisation was >0.82 (Figure 3).

#### Probability of extinction

**Figure 4:**
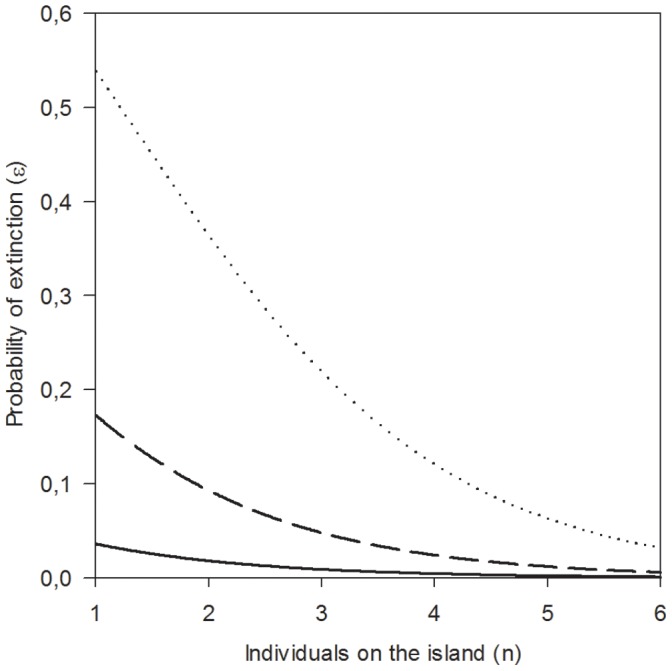
Probability of extinction. The relationship between the probability of extinction (ε), population density index on the island the previous season (POPISL) and maximum water lever (WMAX; 255 m = solid line, 256 m = broken line, 257 m = dotted line).

The probability of extinction increased with the maximum water level (WMAX) and decreased with population density on the island (POPISL; Table 2). Habitat quality (PFOOD), which was a significant univariate predictor of extinction probability, did not enter the best multivariate model possibly because it was correlated with local population density (POPISL). When a high flood had occurred (WMAX = 257 m asl), islands with low density (POPISL = 2; corresponding to 8 individuals when 4 surveys were conducted) had an extinction probability of 0.36. The probability of extinction decreased to 0.09 if the flood only reached medium levels (WMAX = 256), and at low flood levels (WMAX = 255) the extinction probability was only 0.01 (Figure 4).

## Discussion

The studied metapopulation of root voles showed extensive occupancy dynamics. Nevertheless, over time colonisation balanced extinction in the presence of a large mainland source population and there was no trend in occupancy rates which balanced around 50% from the start to the end of the study (i.e. the metapopulation was stationary). However, the temporal variability was considerable; in some seasons nearly all islands were occupied, while in other seasons nearly all islands were extinct. Temporal predictors of the vital rate parameters, not included in classical metapopulation theory, such as spatially correlated environmental stochasticity (i.e. river dynamics) and regional population dynamics (i.e. mainland population size) was needed to take into account this temporal variability. Moreover, habitat quality was a better spatial predictor (PFOOD) than geometric predictors such as island size and isolation covariates.

Accounting for imperfect detection was necessary to achieve unbiased estimates of the occupancy dynamics in our study. Previous studies with the same kind of traps (Ugglan) found individual capture probability >90% of root voles populations on small habitat patches in enclosures and with a higher trap density than in the present study [41]. In open natural populations trapping probabilities are usually lower [27], underlining the need for correcting for detection probability even at the population level. Moreover, the detection probability which averaged 70% in our study needed to be modelled as function of season and size of the island. Detection peaked in the middle of summer and on the largest islands it was close to 90%. This indicates that detection is determined by the number of trappable voles on the island and that some proxy of population size should be included when modelling detection. Without accounting for the effect of population size on detection rate the role of small habitat patches in metapopulation persistence may be underrated [39], which may have serious implications for management and conservation.

The probability of colonisation was not related to island geometry as previously found in many studies of species living in highly fragmented landscapes [2], nor was this rate parameter affacted by river dynamic. When individuals entered the water it is likely that they were carried away by the strong currents and evidently ended up at any random downstream island (or the mainland). Alternatively, root voles may be such good swimmers that the range of distance over water in the present study was smaller than the swimming capacity of the species (cf. [42] for a documentation of swimming capacity of the related field vole). One could have expected that larger islands would receive more individuals just by chance (i.e. because they are large [25]). In our study this was apparently not the case. In agreement with an increasing number of studies, we found a strong impact of habitat quality on colonisation probability [8], [11]. Direct assessment of habitat quality by voles can only occur after arrival on islands. Previous studies of enclosed rote vole populations have demonstrated the importance of food and cover for root vole habitat selection [22], [43]. Individuals arriving at the islands of lowest habitat quality may have rejected the whole island as suitable for settlement and entered the water again in search for better habitats. In particular, this is to be expected if the cost of swimming is low. Hence, it is reasonable to assume that root voles arrived at larger island more frequently than smaller islands just by chance, but that their preference for high quality habitats was strong enough to overcome the risks associated with searching for better habitats on other islands. Alternatively, individuals arriving on these islands of low habitat quality may have been more likely to die in advance of the next trapping session (i.e. they were never captured).

The probability of colonisation was strongly related to the number of individuals on the mainland. Thus in the presence of a temporally large source population, providing a large number of mainland migrants likely to enter the water, the probability of island colonisation peaked [16], [44]. The flow of individuals from the mainland was probably necessary to secure the long-term balance between extinction and colonisation in our study. Hence, the present study system could be characterized as a source-sink or a mainland-island metapopulation [2], [14]. If the study system reflects the essential spatial dynamics of cyclically fluctuating vole populations, where a few large source populations subsidizes smaller patches by providing colonists at peak densities [45], the recent dampening of such cycles observed in boreal and arctic regions [46], [47] is also likely to alter the habitat distribution of voles and lemmings.

Local population density and maximum water level had strong impact on the probability of extinction, while none of the island geometry covariates provided any additional predictive power. This is in agreement with recent studies proving that patch area may not always be a proper surrogate of population size [9], [10]. In addition, our results confirm that dynamic features of the landscape, representing spatially correlated environmental stochastcity, may substantially influence on extinction [17]. The causal relation between extinction risk and maximum water level was most likely inundation of islands. We were not able to continually measure the degree of inundation on island during floods. However, at the highest water level 72% of all traps were washed away from the islands, demonstrating that a substantial proportion of the habitable area of the islands was affected by flooding. Patch destruction will obviously affect extinction [17]. However, in our study the islands were not destroyed, but regained its suitability after withdrawal of flood waters. As a regular “inhabitant of seasonally flooded land” [26] the root vole may be expected to be well adapted to such temporal disturbances.

### Conclusion

The present study of root voles in the specific setting of a riparian mainland-island metapopulation has provided insights that are likely to have general implications for our understanding of factors that rule the spatio-temporal dynamics of fluctuating vole populations. The regionalized multi-annual dynamics of such populations, that often exhibit cyclicity, is a very strong predictor of the local colonisation-extinction dynamics. At peak densities large source populations have the potential to provide colonists even to remote habitat patches imbedded in hostile matrix areas. Owing to high capacities for dispersal and habitat tracking voles can rapidly colonise high quality habitat patches across the entire landscape that previously harboured local populations that have gone extinct due to demographic (small population size) or environmental stochasticity (e.g. extreme climate events). Voles also appear to be more vulnerable to habitat changes that involve deterioration of quality rather than changes in geometric features (e.g. habitat fragmentation).
